# Incidence of Acute Kidney Injury and Mortality Post Successful Cardiac Surgery in a Kashmiri Cohort: A Prospective Comparison of the RIFLE and KDIGO Criteria

**DOI:** 10.7759/cureus.67453

**Published:** 2024-08-22

**Authors:** Hadiya Jan, Chetan Ram, Mohammad A Bhat, Farooq A Ganie, Manmohan Singhal, Mandeep K Arora

**Affiliations:** 1 Faculty of Pharmacy, School of Pharmaceutical and Population Health Informatics, DIT University, Dehradun, IND; 2 Pharmacology, Faculty of Pharmacy, School of Pharmaceutical and Population Health Informatics, DIT University, Dehradun, IND; 3 Nephrology, Sheri Kashmir Institute of Medical Sciences, Srinagar, IND; 4 Cardiovascular Thoracic Surgery, Sheri Kashmir Institute of Medical Sciences, Srinagar, IND

**Keywords:** kdigo criteria, rifle criteria, post-cardiac surgery, mortality, acute kidney injury

## Abstract

Background and objectives

In critically ill patients, acute kidney injury (AKI) influences mortality and morbidity. Few studies have looked at mortality and the frequency of AKI following successful heart and thoracic operations. The current study investigates the association between AKI and mortality rates among patients undergoing post-cardiac surgery care within the Cardiology & Cardio Vascular Thoracic Surgery (CVTS) Intensive Care Unit (ICU).

Methodology

In this prospective research, 124 patients who underwent successful cardiovascular and thoracic procedures between June 2022 and June 2023 were admitted to the CVTS ICU. To determine mortality, we contrasted the two scoring methods, Kidney Disease-Improving Global Outcomes (KDIGO) and Risk, Injury, Failure, Loss of kidney function, and End-stage kidney disease (RIFLE).

Results

Based on the KDIGO criteria, AKI was identified in 37.90% (n = 47) of the patients, and it was identified in 15.32% (n = 19) of the patients utilizing RIFLE. Notably, patients diagnosed with AKI using either the RIFLE criteria or KDIGO criteria exhibited considerably higher mortality rates (p< 0.001). Receiver operating characteristic (ROC) analysis demonstrated the effectiveness of both scoring systems in identifying mortality (area under the ROC curve for RIFLE = 0.224 and KDIGO = 0.150).

Conclusion

Post-cardiac surgery, AKI escalates both mortality and morbidity rates. Despite KDIGO detecting more severe renal injury and mortality, both scoring systems exhibit comparable sensitivity and specificity in predicting death among patients undergoing various cardiovascular and thoracic procedures.

## Introduction

Acute kidney injury (AKI) is characterized by a sudden decline in kidney functionality, resulting in the accumulation of waste products, electrolyte imbalance, and changes in fluid volume. Age, comorbid conditions, nephrotoxic exposure, proteinuria, major surgical procedures, sepsis, fluid resuscitation, and volume status are considered risk factors for AKI. There is compelling evidence that the incidence of AKI is rising rapidly, particularly among patients who are hospitalized with acute illnesses and those undergoing major surgery, particularly cardiac surgeries [1]. It has been reported that the overall mortality rate for individuals undergoing cardiac surgery may be as high as 8%, and postoperative acute kidney injury may elevate the mortality rate to over 60%. In patients undergoing dialysis, the mortality rate ranges from 25% to 88.9%, making postoperative AKI an independent risk factor associated with mortality that results in an eight-fold rise in the risk of death [2]. It is worthwhile to note that the incidence of AKI in patients undergoing cardiac surgery may increase the mortality rate, ranging from 0.4-4.4% to 1.3-22.3% [3]. Even survivors of AKI, especially those considered for renal replacement therapy, have a lower quality of life and require significantly more health care than the general population due to longer hospitalizations, urgent intensive care unit admissions, and re-hospitalizations [4]. Additionally, cardiac surgery is more associated with severe complications, extended intensive care, and a decline in life quality. Additionally, it is associated with early and late mortality, along with higher healthcare costs [5].

Acute kidney injury (AKI) following cardiac surgery has been associated with a variety of factors, including advanced age, male gender, obesity, valve replacement procedures, reduced cardiac output, recent myocardial infarction, extended use of cardiopulmonary bypass, administration of inotropic or vasoconstrictor agents, blood transfusions, intra-aortic balloon pump usage, chronic obstructive pulmonary disease, heart failure, diabetes mellitus, chronic renal disease, and peripheral vascular disease [6-8]. Early identification of individuals at risk of AKI following cardiac surgery is critical for improving their care both intraoperatively and postoperatively. Research indicates that even slight postoperative rises in serum creatinine (SCr) levels are strongly associated with a substantial increase in the risk of mortality [9]. It is worthwhile to note that the Acute Dialysis Quality Initiative (ADQI) group, with the objective of studying the AKI, emphasized classification for the AKI definition [10]. The Risk, Injury, Failure, Loss of kidney function, and End-stage kidney disease (RIFLE) classification system is founded upon the determinants of serum creatinine (SCr) and urine output (UO). It categorizes AKI into three severity classes, i.e., risk, injury, and failure, based on the changes in SCr and/or UO. Additionally, it classifies AKI into two outcome classes (loss of kidney function and end-stage renal disease). The patient's classification should be determined based on the criteria of SCr and/or UO that result in the most severe classification, specifically the maximum RIFLE classification. For example, if a patient falls into the risk class based on UO but the injury class based on SCr variation, the more severe criteria, i.e., SCr, should be utilized to determine the severity of AKI in the patient [11]. Furthermore, the Kidney Disease-Improving Global Outcomes (KDIGO) clinical practice recommendations introduced an entirely new categorization, AKI. According to the KDIGO 2012 guidelines, AKI is characterized by a rise in SCr levels of at least 0.3 mg/dL over 48 hours or a 50% increase in SCr within the preceding 7 days [12]. The staging system was preserved in accordance with the Acute Kidney Injury Network (AKIN) guidelines, with the inclusion of a glomerular filtration rate (GFR) threshold of less than 35 mL/min/1.73 m^2^ for pediatric patients as an additional criterion for stage 3 AKI [13].

When combined, the definitions used to estimate the occurrence of AKI following cardiac surgery range from 5% to 42% in different contexts and geographical areas. There are two commonly used consensus definitions for the diagnosis of AKI in contexts other than cardiac surgery: RIFLE and KDIGO. However, there is currently no agreed criteria or consistent criterion for AKI in post-cardiac surgery patients [14]. A common definition for identifying and categorizing AKI would make it easier to manage these patients' conditions and improve their outcomes. In terms of comparing these classifications' ability to identify AKI and forecast results in the Indian population, there is little evidence available. To precisely estimate the degree of AKI and design the best management measures while keeping in mind the advantages and disadvantages of each criterion, this comparison is of paramount importance. Thus, the purpose of this study was to examine the RIFLE and KDIGO AKI criteria to determine the frequency of AKI and their prognostic capacity for all-cause mortality and morbidity following successful heart surgery.

## Materials and methods

**Figure 1:**
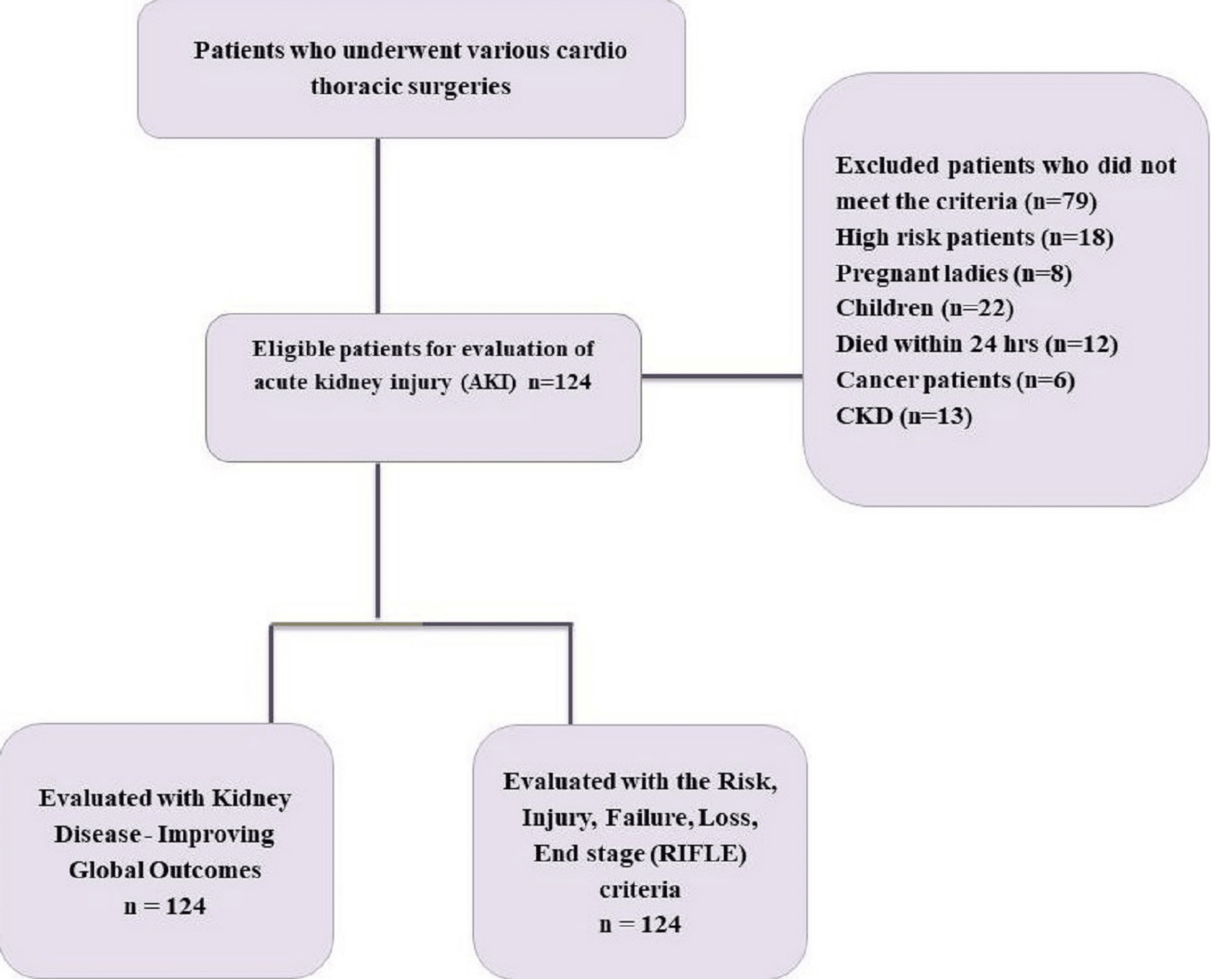
Flow diagram for the patient selection for comparison to acute kidney injury. KDIGO: Kidney Disease-Improving Global Outcomes; RIFLE: Risk, Injury, Failure, Loss of kidney function, and End-stage kidney disease

This prospective observational study was conducted in the ICU at Sheri Kashmir Institute of Medical Sciences (SKIMS), Jammu and Kashmir, India. Ethical permission was obtained from the University Clinical Studies Ethics Committee and Medical Institute Ethical Committee DIT University, Dehradun, India, under protocol number DITU/UREC/2022/04/3. A total of 143 patients who underwent various cardiovascular and thoracic surgeries between June 1, 2022, and June 30, 2023, were prospectively evaluated. The available intensive care unit is a cardiovascular and thoracic surgery intensive care unit that receives patients undergoing different types of cardiovascular and thoracic surgeries. The common CTS procedures used in our study include coronary artery bypass grafting (CABG), aortic valve replacement (AVR), mitral valve replacement (MVR), double valve replacement (DVR), thoracic endovascular aortic repair (TEVAR), lobectomy, and esophagectomy. All individuals were previously admitted to the medical facility before undergoing cardiac surgery, and their progress was monitored until they were discharged or experienced mortality. The demographic and clinical information of the patients was obtained and documented prospectively. Patients with pre-existing renal ailments, end-stage malignancies, individuals below 18 years of age, pregnant women, individuals with chronic kidney disease (CKD), those who had undergone iodinated contrast administration within 72 hours before surgery (due to associated kidney-related risks), and patients with preoperative eGFR <60 ml/min were excluded from the study. Additionally, individuals who experienced mortality within the initial 24 hours following surgery were also excluded. Data of the other 124 individuals were collected from the patient files at the ICU. Figure 1 displays a comprehensive flow diagram outlining the investigation in detail. Both the RIFLE-based classification and the KDIGO-based classification were employed to evaluate all patients.

By incorporating the Stage L: RIFLE criteria for kidney function loss and Stage E: end-stage renal failure into Stage F: the failure group), this three-stage system consistent with KDIGO was established for assessing the diagnostic and staging capabilities of both scoring systems. The patients' follow-up records provided information on the patient's sex, age, urine output, SCr levels, and renal replacement treatment. Patients were deemed survivors if they lived for 30 days following surgery. Survival and mortality data were collected for survivors and non-survivors using daily follow-up forms, assessing creatinine levels and UO daily according to RIFLE and KDIGO standards from the first admission. Our main goal was to evaluate the two methods' rates of renal failure identification and the 30-day mortality rates for patients who had the condition in each categorization. Additionally, a secondary analysis compared survivors and non-survivors regarding age, sex, creatinine levels, co-morbidities, and smoking habits.

**Figure 2:**
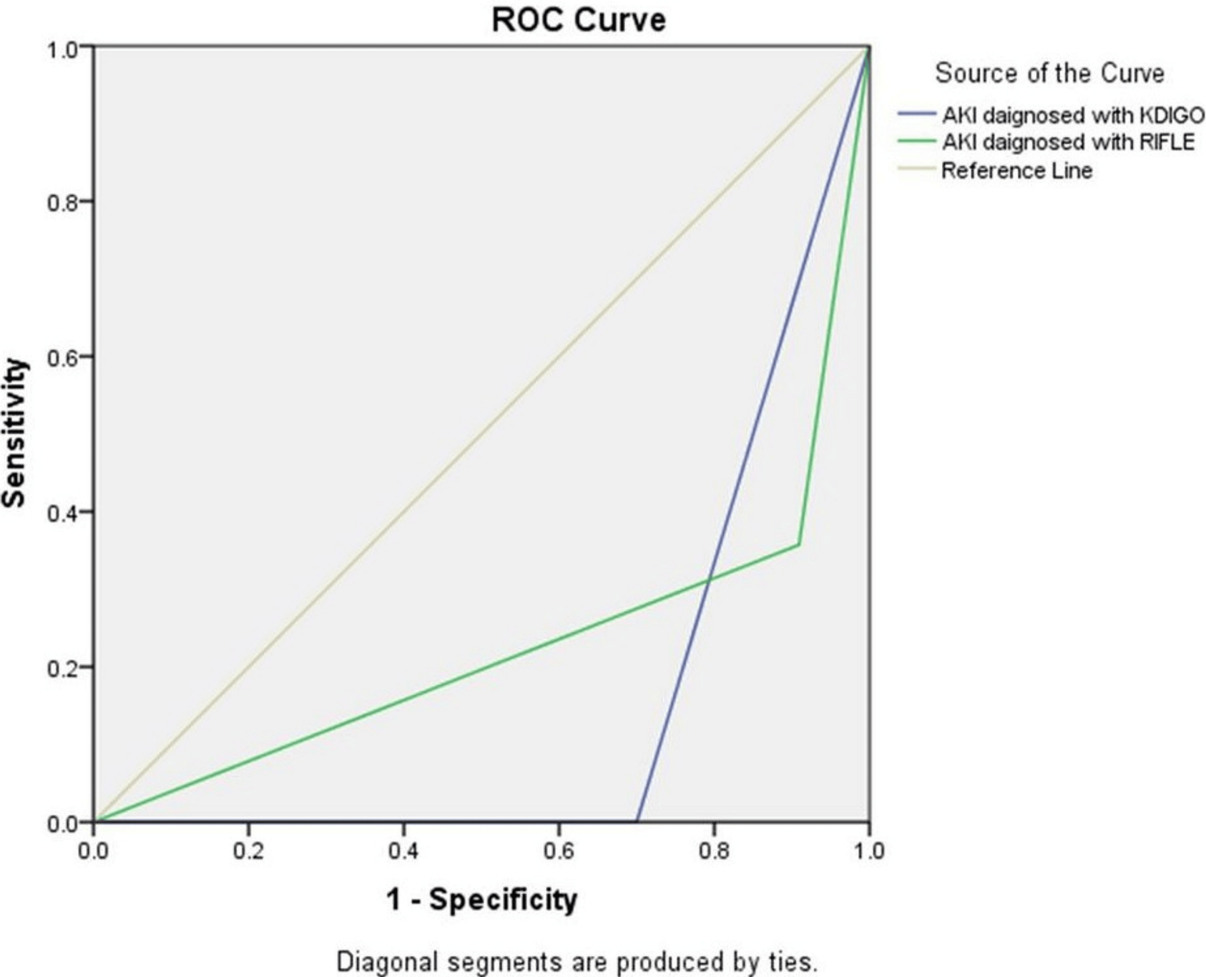
Receiver operating characteristics (ROC) curve for predicting mortality in RIFLE and KDIGO Area under curve AKI diagnosed with KDIGO: 0.150 (p = 0.001) and AKI diagnosed with RIFLE: 0.224 (p = 0.001). ROC curve analysis revealed a considerable difference between the two systems. AKI: Acute Kidney Injury; KDIGO: Kidney Disease-Improving Global Outcomes; RIFLE: Risk, Injury, Failure, Loss of kidney function and End-stage kidney disease

The sample size calculation utilized the ClinCalc tool (https://clincalc.com/stats/samplesize.aspx). To achieve 95% power with a Type I error rate based on our pilot trial results, which included 15 renal damage patients, a total of 124 patients were required. Among these, 50% of RIFLE-diagnosed patients and 70% of KDIGO-diagnosed patients experienced mortality. The software from the Statistical Package for Social Sciences (SPSS) 16.0 program (SPSS Inc. Released 2007. SPSS for Windows, Version 16.0. Chicago, SPSS Inc.) was utilized for the statistical analysis. Mean and standard deviation were utilized as descriptive statistics for parameters with regularly distributed distributions. Number and percentage values were used to express categorical variables. The Mann-Whitney U test was used to assess the differences between two groups for people with non-normal distributions. To assess the association between categorical variables, the chi-square test was applied. All other variables, except for gender, are independent. The variables happen with equivalent probabilities. The predicted accuracy of the two-scoring system mortality was further evaluated using receiver operating characteristic (ROC) analysis. A p-value < 0.05 was considered statistically significant (Figure 2).

## Results

A total of 124 patients were part of this research as depicted in Figure 1; the results are shown in Table 1. The average age of patients was found to be 47±15.51 (n=124); the majority (51.61%, n=64) were males; 20.16% (n=25) were active smokers; and 16.13% were ex-smokers. Around 18% (n=22) of patients were diagnosed with hypertension, 7.26% (n=9) with diabetes, and 8.06% (n=10) with thyroid. Of the total operated patients, approximately 38% (n=47) suffered from AKI diagnosed with KDIGO, and 15.32% (n=19) from AKI diagnosed with RIFLE criteria. The mortality rate was found to be 11.29% (n=14) in patients diagnosed with AKI.

**Table 1:** Patient characteristics and clinical data (n=124) AKI; Acute Kidney Injury; KDIGO: Kidney Disease-Improving Global Outcomes; RIFLE: Risk, Injury, Failure, Loss of kidney function, and End-stage kidney disease

Age (Years)	47±15.51
Sex (Male/Female)	64/60(51.61% vs. 48.39%)
Active smokers	25 (20.16%)
Ex-smokers	20 (16.13%)
Hypertension	22 (17.74% )
Diabetes	9( 7.26% )
Thyroid	10(8.06%)
AKI diagnosed with KDIGO	47 (37.90 %)
AKI diagnosed with RIFLE	19 (15.32% )
Non-survivors	14 (11.29% )

The Mann-Whitney U test was used to check the significant difference in demographic and clinical data between survivor and non-survivor patients as presented in Tables 2, 3. The results revealed that there was a significant difference between survivor and non-survivor patients in co-morbidities like diabetes, thyroid, AKI diagnosed with KDIGO, and AKI diagnosed with RIFLE criteria. 

**Table 2:** Comparison of demographic data of survivor and non-survivor patients ⁕Significance at 5 % level; P value is a statistical measure that indicates whether or not an effect is statistically significant.

Demographics	Survivors	Non Survivors	P value
Age	47.25	53.29	0.141
Male	57 (51.8 %)	7 (50.0 %)	0.898
Female	53 (48.2 %)	7 (50.0 %)	

**Table 3:** Comparison of clinical data of survivor and non-survivor patients ⁕Significance at the 5% level; P value is a statistical measure that indicates whether or not an effect is statistically significant. AKI; Acute Kidney Injury; KDIGO: Kidney Disease-Improving Global Outcomes; RIFLE: Risk, Injury, Failure, Loss of kidney function, and End-stage kidney disease

Variables	Survivors		Non Survivors		P value
Active smokers	Yes	No	Yes	No	N/A
Ex-smokers	23 (20.9%)	87 (79.1%)	2 (14.3%)	12 (85.7%)	0.562
Smokers	19 (17.3%)	100 (82.7%)	1 (7.1%)	13 (92.9%)	0.334
Hypertension	19 (17.3%)	100 (82.7%)	3 (21.4%)	11 (78.6%)	0.703
Diabetes	5 (4.5%)	105 (95.5%)	4 (28.6%)	10 (71.4%)	0.001*
Thyroid	9 (8.2%)	100 (91.8%)	1 (7.1%)	13 (92.9%)	0.893
AKI diagnosed with KDIGO	33 (30.0%)	77 (70.0%)	14 (100.0%)	0 (0.0%)	0.0001*
AKI diagnosed with RIFLE	10 (9.1%)	100 (90.9%)	9 (64.3%)	5 (35.7%)	0.0001*

The average age of AKI patients diagnosed with KDIGO was 49.63±15.08 and AKI diagnosed with RIFLE was 50.78±13.06. Around 46.8% (n=22) of patients with KDIGO and 42.10% (n=8) of patients with RIFLE were male. Around 21% (n=10) of patients diagnosed with KDIGO were active smokers, and 8.5% (n=4) were ex-smokers. While 21% (n=4) were found to be active smokers and 10.5% (n=2) were ex-smokers in patients diagnosed with RIFLE criteria, around 21.27% (n=10) were diagnosed with hypertension, 12.76% (n=6) with diabetes, and 12.76% (n=6) with thyroid in patients with KDIGO. Around 15.78% (n=3) suffered from hypertension, 5.26% (n=1) from diabetes, and 10.52% (n=2) from thyroid in patients diagnosed with RIFLE criteria (Table 4). The mortality rate was found to be 29.78% (n=14) in patients diagnosed with KDIGO and 47.36% (n=9) in patients diagnosed with RIFLE criteria.

**Table 4:** Comparison of AKI patients' mortality, smoking, and comorbid conditions based on the RIFLE and KDIGO criteria AKI; Acute Kidney Injury; KDIGO: Kidney Disease-Improving Global Outcomes; RIFLE: Risk, Injury, Failure, Loss of kidney function, and End-stage kidney disease

Variables	AKI diagnosed with KDIGO	AKI diagnosed with RIFLE
Age	49.63±15.08	50.78±13.06
Sex (Male/Female)	22/25(46.8% vs. 53.19%)	8/11 (42.10% vs. 57.89%)
Active smokers	10 (21%)	4 (21%)
Ex-smokers	4 (8.5%)	2 (10.5%)
Hypertension	10 (21.27%)	3 (15.78%)
Diabetes	6 (12.76%)	1 (5.26%
Thyroid	6 (12.76%)	2 (10.52%)
Mortality	14 (29.78%)	9 (47.36%)

## Discussion

The study revealed a relationship between kidney impairment, evaluated using the RIFLE and KDIGO criteria, and the fatality rate after heart surgery. However, a greater proportion of patients had renal damage diagnosed when the KDIGO criteria were used as opposed to RIFLE. According to KDIGO, 37.90% (n = 47) of patients had AKI as compared to 15.32% (n = 19) according to RIFLE. These findings lend support to both the RIFLE and KDIGO categories in the existing literature. AKI is associated with high morbidity and fatality, as well as prompt identification of heart surgical complications. AKI is crucial for alerting doctors to the possibility of renal injury and enabling prompt intervention.

The study revealed a connection between kidney impairment, evaluated using the RIFLE and KDIGO criteria, and the mortality rate after heart surgery. However, when the KDIGO criteria were used instead of the RIFLE criteria, a higher percentage of individuals were determined to have renal damage. Based on KDIGO criteria, 37.90% (n = 47) of the patients had AKI, as opposed to 15.32% (n = 19) according to the RIFLE criteria. The RIFLE and KDIGO categories in the literature are both supported by these findings [15].

AKI is linked to severe morbidity and mortality, and the detection of post-cardiac surgery risk for AKI is crucial because it makes the physician more aware of the possibility of renal injury and enables prompt intervention and therapy.

The RIFLE criteria were created in 2004 to standardize the definition of AKI [9,16]; however, it was changed soon after by the AKIN [16-18] when it emerged that even minor variations in SCr might be linked to higher mortality. The AKIN criterion classified these minute fluctuations in SCr values over 48 hours to identify the change in creatinine. Then, in 2012, the KDIGO AKI study team updated the definition of AKI to include changes in creatinine within 48 hours or a decline in GFR over seven days [19]. The KDIGO recommendations define AKI as a rise in SCr concentration of 0.3 mg/dL or more within 48 hours. The aforementioned definitions of AKI have been independently verified in numerous investigations involving the general population and are therefore commonly used [20]. To forecast the level of AKI and unfavorable outcomes, including the need for KRT, morbidity, and death in patients undergoing cardiac surgery, each defining system has advantages and limitations of its own. As a result, efforts are currently being made to identify the definition of AKI following cardiac surgery that will have the biggest influence on the result.

Both approaches are efficient at estimating the risk of mortality, according to the ROC analysis. Based on the KDIGO criteria, 80.5% of the patients in Kim et al.'s retrospective review of 82 cardiac arrest episodes had AKI [21]. AKI, or failure, was determined in 33 (31.4%) of the 105 cardiac arrest patients in a study by Chua et al. using the RIFLE criteria [22]. AKI staging was done in the aforementioned trials within the first 24 to 48 hours after ICU admission, and the changes in AKI severity during ICU follow-up were not examined. The AKI stage was determined in our study by taking into consideration the patient's highest creatinine level and/or lowest UO during the first week of ICU follow-up, which represented changes that took place during follow-up.

In light of the available research and our findings, it is also possible to conclude that the KDIGO classification provides a more accurate prediction of death than RIFLE (mortality in patients identified using RIFLE vs. KDIGO: 47.36% vs. 29.78%), in addition to having a higher sensitivity for identifying AKI [23].

Patients who experienced AKI had a greater mortality rate (65%) than those who did not (50%), according to Tujjar et al. [24]. According to the literature, renal damage measured using both the RIFLE and KDIGO criteria and death were significantly correlated in our study. Geri et al. investigated the association between the progression of AKI and mortality rate in 580 patients who underwent out-of-hospital cardiac arrest and found that stage 3 kidney injury was related to a considerably greater mortality rate [25]. In our investigation, the degree of renal injury was correlated with a progressive increase in mortality rate. According to Yanta et al., the physiological function of the kidneys decreased with advancing age, which was associated with the progression of AKI after cardiac arrest [26]. In the course of our research, we also discovered that patients with AKI as defined by the RIFLE and KDIGO criteria had mean ages that were higher than those of patients without AKI. Patients with KDIGO diagnoses had a mean age of 49.63±15.08 while those with RIFLE diagnoses had a mean age of 50.78± 13.06.

The initial post-cardiac arrest hospitalization mean creatinine level, according to Hasper et al., was 1.20 mg/dL [27]. In this study, the average level of serum creatinine was 0.87 mg/dL, and KDIGO criteria-based AKI diagnosis was strongly linked to high SCr levels.

Our descriptive findings demonstrated that having concomitant conditions, such as diabetes, hypertension, and thyroid disease, was associated with a higher probability of AKI. Similar relationships have been found by others as well [28-30].

Limitation

Some restrictions should be used when interpreting our results. Because the study was carried out at a single center, the sample size was insufficient for assessments other than the primary outcome. It’s possible that the conclusions cannot be generalized. We provided rough estimations that may have exaggerated the severity of the AKI in the given cohort because they were not corrected for coexisting diseases. Additionally, we did not define AKI using the criteria for urine output. Additional risk factors for AKI include chronic obstructive pulmonary disease, congestive heart failure, female gender, prior cardiac surgery, the urgency of the procedure, and a reduced left ventricular ejection fraction of less than 35% [28]. However, because we did not take into account or examine these factors for our investigation, we did not detect such a correlation in our study group.

## Conclusions

In conclusion, mortality rates after various kinds of cardiac procedures raise the risk of AKI. The efficacy of the two techniques in predicting death in patients after cardiac surgery is comparable; however, KDIGO criteria can detect more renal damage and mortality than RIFLE criteria. More research is needed to validate the best method for predicting patient mortality after successful cardiac, vascular, and thoracic surgeries.
